# How appropriate is treating patients diagnosed with advanced esophageal cancer with anticancer drugs? A multicenter retrospective cohort Spanish study

**DOI:** 10.1007/s12094-024-03436-1

**Published:** 2024-04-25

**Authors:** Marilina Santero, Carolina Requeijo, Maria Jesus Quintana, Dulce Rodríguez, David Bottaro, Ismael Macias, Carles Pericay, Natalie Farina, Jesus Manuel Blanco, Iratxe Urreta-Barallobre, Laura Punti, Maria Angeles Nava, Xavier Bonfill Cosp

**Affiliations:** 1 Department of Paediatrics, Obstetrics, Gynaecology and Preventive Medicine and Public Health, Universitat Autònoma Barcelona (UAB), Barcelona, Spain; 2 Institut de Reserca Sant Pau (IR SANT PAU), Barcelona, Spain; 3 Centro de Investigación Biomédica en Red de Epidemiología y Salud Pública (CIBERESP), Madrid, Spain; 4 Hospital Universitari Sant Joan de Reus, Reus, Spain; 5 Corporació Sanitària Parc Taulí, Barcelona, Spain; 6 Hospital Universitario Donostia, Donostia, Spain; 7 Clinical Epidemiology, Biodonostia Health Research Institute, Donostia-San Sebastián, Spain; 8 Clinical Epidemiology Unit, Osakidetza Basque Health Service, Donostia University Hospital, Donostia-San Sebastián, Spain; 9 Hospital de Mataró, Mataró, Spain; 10 Centro Cochrane Iberoamericano, Barcelona, Spain; 11 Hospital de la Santa Creu i Sant Pau, Barcelona, Spain

**Keywords:** Advanced esophageal cancer, Systemic oncological treatment, Appropriateness, Palliative care

## Abstract

**Aim:**

To assess the appropriateness of systemic oncological treatments (SOT) provided to patients diagnosed with advanced esophageal cancer (EC) across a group of participating hospitals.

**Methods:**

Multicenter, retrospective cohort study in five Spanish hospitals including newly confirmed advanced EC cases between July 1, 2014, and June 30, 2016, with a 5-year follow-up.

**Results:**

We identified 157 patients fulfilling the inclusion criteria (median age: 65 years, 85.9% males). Most patients, 125 (79.6%) were treated at least with one active treatment, and 33% received two or more lines of SOT. The 1-, 2- and 5-year overall survival rates were 30.3% [95%CI: 23.8, 38.7], 14.0% [95%CI: 9.3, 21.0], and 7.1% [95% CI: 3.8, 13.1] respectively, and the median survival time 8 months (95% CI: 6, 19) for stages IIIb IIIc and 7 months (95% CI: 5, 9) for stage IV. Clinical stage, receiving more than one line of SOT, and treatment with radiotherapy accelerated the time to death (0.4, 0.9-, and 0.8-times shorter survival respectively, p < 0.05). Better performance status (ECOG < 2) extended survival time by 2.2 times (p = 0.04). Age < 65 years (OR 9.4, 95% CI 3.2, 31.4, p < 0.001), and being treated in one particular hospital (OR 0.2, 95% CI 0.0, 0.8, p < 0.01) were associated with the administration of two or more lines of SOT. Altogether, 18.9% and 9.0% of patients received chemotherapy in the last four and two weeks of life, respectively. Moreover, 2.5% of patients were prescribed a new line of chemotherapy during the last month of life. The proportion of all patients who did not have access to palliative care reached 29.3%, and among those who had access to it, 34.2% initiated it in the last month of life.

**Conclusion:**

A high proportion of advanced EC patients receive many treatments not based on sound evidence and they do not benefit enough from palliative care services. The most accepted appropriateness indicators point out that some of the analyzed patients could have been overtreated. This study provides important insights into the quality of care provided to advanced EC, and furthermore, for giving valuable insight and opportunities for improvement.

## Introduction

Esophageal cancer (EC) is a highly prevalent disease, ranking eighth in cancer incidence worldwide, with over 600,000 new cases diagnosed in 2020, of which 70% are in men [1, 2]. Unfortunately, it is often not detected until advanced or metastatic stages, resulting in a very poor survival rate (ratio mortality to incidence 0.88), even in high-income countries [1]. In fact, EC ranks sixth in cancer mortality, with over half a million deaths reported globally in 2020. In Spain alone, 1,823 deaths were attributed to this disease [3]. Despite significant advancements in medical research and treatment options, age-standardized 5-year net survival rates for the most advanced stages remain dismally low, with less than 10% survival reported in some studies [4–6].

In the 1990s, multiple studies investigating the most cytotoxic chemotherapy combinations led to the adoption of combined cisplatin and 5-fluorouracil (5-FU) as the standard of care for treating recurrent or metastatic stages of EC by the U.S. Food and Drug Administration (FDA) [7]. This treatment regimen was associated with a median survival of 12.7 months (95% CI 11.9, 13.5 months) for the 5-fluorouracil plus cisplatin group [8]. Over the past two decades, a range of new targeted therapies and immunotherapy have emerged and are now being used progressively for the treatment of advanced EC patients [9–13]. Nonetheless, their efficacy is still limited, and their use is associated with a high incidence of adverse events and toxicities [9–11].

The poor short-term prognosis for patients with advanced EC, with most dying within a year of diagnosis, has led some authors to consider this period as an end-of-life (EOL) stage [14, 15]. This presents a significant challenge from both clinical and public health perspectives, as the limited effectiveness of available treatments, potential side effects, and associated costs must be weighed against the short remaining lifespan of the patient. It is well-established that aggressive medical interventions during the final stages of life can have a detrimental impact on the patient's quality of life [16, 17]. Despite this, the use of systemic oncological treatments (SOT) near the EOL period is expanding, leading some experts to warn that the treatment of advanced cancer patients is becoming too aggressive and potentially harmful [18–21]. Moreover, these intensive practices may also delay patients´ referral to palliative care, which can provide essential physical, emotional, and spiritual support during the EOL period [22–24].

Recent studies have attempted to evaluate the appropriateness of treatment in advanced cancer and proposed indicators for the quality of EOL care [25, 26]. However, the appropriateness of SOT has not been well-studied in advanced EC, and furthermore, understanding local practices may provide some opportunities for improvement. Thus, the aim of this study is to assess the appropriateness of SOT provided to patients diagnosed with advanced EC across a group of participating hospitals.

## Methods

### Study design and setting

This study is part of a larger project called the ASTAC-Study, which aims to describe and assess the available evidence on the effectiveness and appropriateness of SOT in advanced non-intestinal digestive cancers [27–32]. To achieve this, we conducted a multicenter, retrospective cohort study in five Spanish hospitals: Hospital Santa Creu i Sant Pau in Barcelona (coordinator), Consorci Sanitària Parc Taulí in Sabadell, Hospital Universitario Donostia in Gipuzkoa, Hospital Universitari Sant Joan in Reus, Hospital in Mataró. The general characteristics of these hospitals are presented in Appendix 1. To ensure transparency and reproducibility, we registered our research protocol and published it online in the Open Science Framework (OSF) repository prior to beginning the review process [28]. Additionally, we followed the STROBE guidelines for reporting observational studies [33] to ensure accurate and comprehensive reporting of our findings.

### Patients

The study included patients who were newly diagnosed with advanced EC (stages IIIb, IIIc, or IV), including gastroesophageal junction, between July 1, 2014, and June 30, 2016, and had confirmed clinical and pathological reports. Patients with missing information on histopathology and cancer stage, no data in clinical records, or progressions from early stages were excluded. The follow-up period began at the time of diagnosis with any advanced stage, and the study endpoint was defined as the date of death, loss to follow-up, last contact, or end of follow-up time (June 30, 2021).

### Variables

We recorded data across several categories including: (a) Sociodemographic information such as age, sex, and residence; (b) Clinical characteristics such as diagnosis date, histological type and grade, immunohistochemistry, tumor location, stage, extension, comorbidities, and functional status at the time of diagnosis (measured by direct ECOG values or transformed from Karnofsky index); (c) Treatment-related variables such as type of treatment prescribed and received (surgery, chemotherapy, radiotherapy, immunotherapy), start date, treatment dates, last cycle, number of chemotherapy lines, and objectives (neoadjuvant, adjuvant, or palliative), participation in research studies, treatment interruption, and causes of toxicity; (d) Follow-up variables including referral to a palliative care or home care unit, date of last contact, vital status, cause of death (if applicable), and place of death.

### Data collection

After conducting a literature review and consulting with clinical experts regarding the most important variables, we developed a custom online questionnaire using the Clinapsis® platform [34], which is designed for the development and management of clinical studies. To evaluate its reliability, we performed a pilot test. Eligible patients were identified based on relevant clinical data from the admission units at the participating hospitals. Data collectors, who were staff from the selected hospitals, recorded baseline and follow-up patient characteristics into a single database after ensuring the validity and completeness of the data. Data collectors received prior training to improve the quality of the data collected.

### Statistical analysis

After ensuring data completeness, we performed descriptive statistics for categorical variables as absolute frequencies and percentages, and for quantitative variables as mean with standard deviation (SD) or median with interquartile range (IQR). For comparative analysis, we used χ^2^ or Fisher’s Exact Test for categorical variables and Student’s t-test for continuous variables. To estimate survival, we used Kaplan–Meier curves and compared them using the two-tailed log-rank test.

A reverse Kaplan–Meier estimator was used to estimate the median follow-up time [35]. Finally, after assessing violations of the proportional hazard assumption in the Cox regression, we chose a lognormal accelerated failure time (AFT) model, a parametric model for the analysis of time-to-event data to estimate the effects of covariates on acceleration/deceleration of the survival time. To do that, we considered a set of ten variables including age, sex, hospital, histology, clinical stage, tumor location, performance status, receiving more than one line of SOT, surgery as the first treatment, and radiotherapy as the first treatment. The results were presented using the exponentiated regression coefficients (i exp (β, time ratio or TR), where TR > 1 for a covariate implies that it prolongs the time to the event, TR = 1 implies no effect, and TR < 1 indicates that the occurrence of an earlier event is more likely. A p-value less than 0.05 was considered statistically significant.

We conducted bivariate analyses to examine associations between sociodemographic and clinical characteristics, and the use of two or more lines of SOT. For continuous variables, t-tests were used, while for categorical variables, χ^2^ tests were employed. Variables that were found to be statistically significant (*p < 0.05*) were included in a multiple logistic regression model to determine which factors were independently associated with the use of two or more lines of SOT. The results are presented as adjusted odds ratios with 95% confidence intervals. Statistical significance was defined as p-values less than 0.05 in the multiple logistic regression model. We conducted all statistical analyses using RStudio Version 1.4.1106 [36].

To evaluate treatment appropriateness, we used a two-phase approach. First, we compared the data collected from hospitals with existing evidence and clinical practice guidelines [30, 37, 38]. Second, we selected indicators based on two major appropriateness dimensions adapted from previously published proposals [25, 30, 31]: (a) overuse of anticancer drugs and (b) underuse of palliative care services. We calculated seven indicators to assess appropriateness: (1) administration of chemotherapy in the last 14 days or (2) in the last 30 days of life, (3) initiation of a new line of chemotherapy in the last 30 days of life, (4) treatment of patients with ECOG performance status ≥ 3 with anticancer drugs, (5) lack of access to any palliative care, (6) admission to palliative care < 30 days before death, or (7) < 3 days before death. For each indicator, we calculated the proportion of patients who met the criteria.

### Ethics

The study was conducted in compliance with the Declaration of Helsinki, and the protocol was approved by the Ethics Committee of all five participating hospitals. Since this was a retrospective study that only used secondary cancer data, patients were not involved in the design or execution of the research. Informed consent was waived because we used anonymized retrospective data.

## Results

A total of 157 newly diagnosed cases of advanced EC were included in the analysis, with a median age of 65 years (range 30–92 years) and 134 (85.9%) male patients. Table 1 summarizes the sociodemographic and general characteristics of the patients.

**Table 1:** General characteristics of advanced esophageal cancer patients at five hospitals, Spain, 2014–2016 (n = 157)

Characteristics	Total (n = 157)	Hospital 1 (n = 31)	Hospital 2 (n = 34)	Hospital 3 (n = 31)	Hospital 4 (n = 41)	Hospital 5 (n = 20)	p value*
Sociodemographic and clinical background							
Age, yr median (IQR)	**65 (15.0)**	68 (17.5)	69 (11.8)	63 (13.0)	62 (13.0)	65 (10.3)	0.149
Men/women (%)	**134/22 (85.9)**	25/6 (80.7)	26/8 (76.5)	25/6 (80.7)	38/3 (92.7)	20/0 (100.0)	**0.042**
Alcohol, n (%)	**50 (34.0)**	16 (53.3)	9 (26.5)	8 (27.6)	14 (41.2)	3 (15.0)	** < 0.001**
Tobacco, n (%)	**57 (38.8)**	10 (33.3)	15 (44.1)	6 (20.7)	17 (50.0)	9 (45.0)	** < 0.001**
BMI, median (IQR)	**24.3 (5.7)**	25.6 (5.8)	23.9 (6.1)	24.6 (3.3)	23.5 (4.7)	24.8 (7.8)	0.543
Comorbidities, n (%)	**117 (76.5)**	29 (93.5)	25 (78.1)	20 (66.7)	25 (78.1)	16 (80.0)	**0.011**
CCI, median (IQR)	**3 (3.0)**	4 (3.5)	4 (2.0)	3 (2.0)	4 (2.0)	4 (3.5)	0.154
ECOG PSº, n (%)							0.296
≤ 2	**139 (88.5)**	25 (80.6)	28 (82.4)	29 (93.5)	39 (95.1)	18 (90.0)	
≥ 3	**15 (9.6)**	4 (12.9)	6 (17.6)	2 (6.5)	1 (2.4)	2 (10.0)	
no data	**3 (1.9)**	2 (6.5)	0	0	1 (2.4)	0	
Anatomopathology primary site, n (%)							0.115
upper	**17 (10.8)**	5 (16.1)	2 (5.9)	5 (16.1)	2 (4.9)	3 (15.0)	
middle	**40 (25.5)**	6 (19.4)	8 (23.5)	6 (19.4)	11 (26.8)	9 (45.0)	
lower	**60 (38.2)**	11 (35.5)	18 (52.9)	9 (29.0)	14 (34.2)	8 (40.0)	
GEJ	**39 (24.8)**	9 (29.0)	6 (17.7)	10 (32.3)	14 (34.2)	0	
Hystology subtype, n (%)							0.085
adenocarcinoma	**78 (49.7)**	19 (61.3)	16 (47.1)	12 (38.7)	24 (58.5)	7 (35.0)	
squamous cell carcinoma	**69 (43.9)**	10 (32.3)	18 (52.9)	14 (45.2)	17 (41.5)	10 (50.0)	
Hystology grade, n (%)							**0.004**
GX	**47 (30.1)**	9 (29.0)	6 (17.6)	13 (41.9)	18 (43.9)	1 (5.0)	
G1	**4 (2.6)**	0	0	3 (9.7)	0	1 (5.0)	
G2	**61 (39.1)**	9 (29.0)	21 (61.8)	7 (22.6)	13 (31.7)	11 (55.0)	
G3	**44 (28.2)**	13 (41.9)	7 (20.5)	7 (22.6)	10 (24.4)	7 (35.0)	
Advanced clinical stage, n (%)							** < 0.001**
IIIb, IIIc	**39 (25.2)**	8 (25.8)	3 (9.4)	13 (42.0)	6 (14.7)	9 (45.0)	
IV	**116 (74.8)**	23 (74.2)	29 (90.6)	18 (58.1)	35 (85.4)	11 (55.0)	

*BMI* body mass index, *CCI* Charlson Comorbidity Index, *ECOG PS* Eastern Cooperative Oncology Group Performance Status, *GEJ* gastroesophageal junction

*t-test for continuous variables and chi-square test for categorical variables; ºKarnofsky scores were transformed to ECOG PS scoring

Adenocarcinomas were slightly more prevalent (n = 78, 49.7%) than squamous cell carcinomas (n = 69, 43.9%). Approximately 75% of cases were classified as stage IV, with metastases mostly in distant lymph nodes, the peritoneal cavity, and the liver, while the remaining 25% were classified as stages IIIb and IIIc. Most cases (n = 139, 88.5%) were classified as having an ECOG performance status equal to or less than two. The hospitals were generally similar in most variables, except for sex, histology grade, clinical stage, tobacco and alcohol use, and comorbidities.

### Treatments

**Fig. 1:**
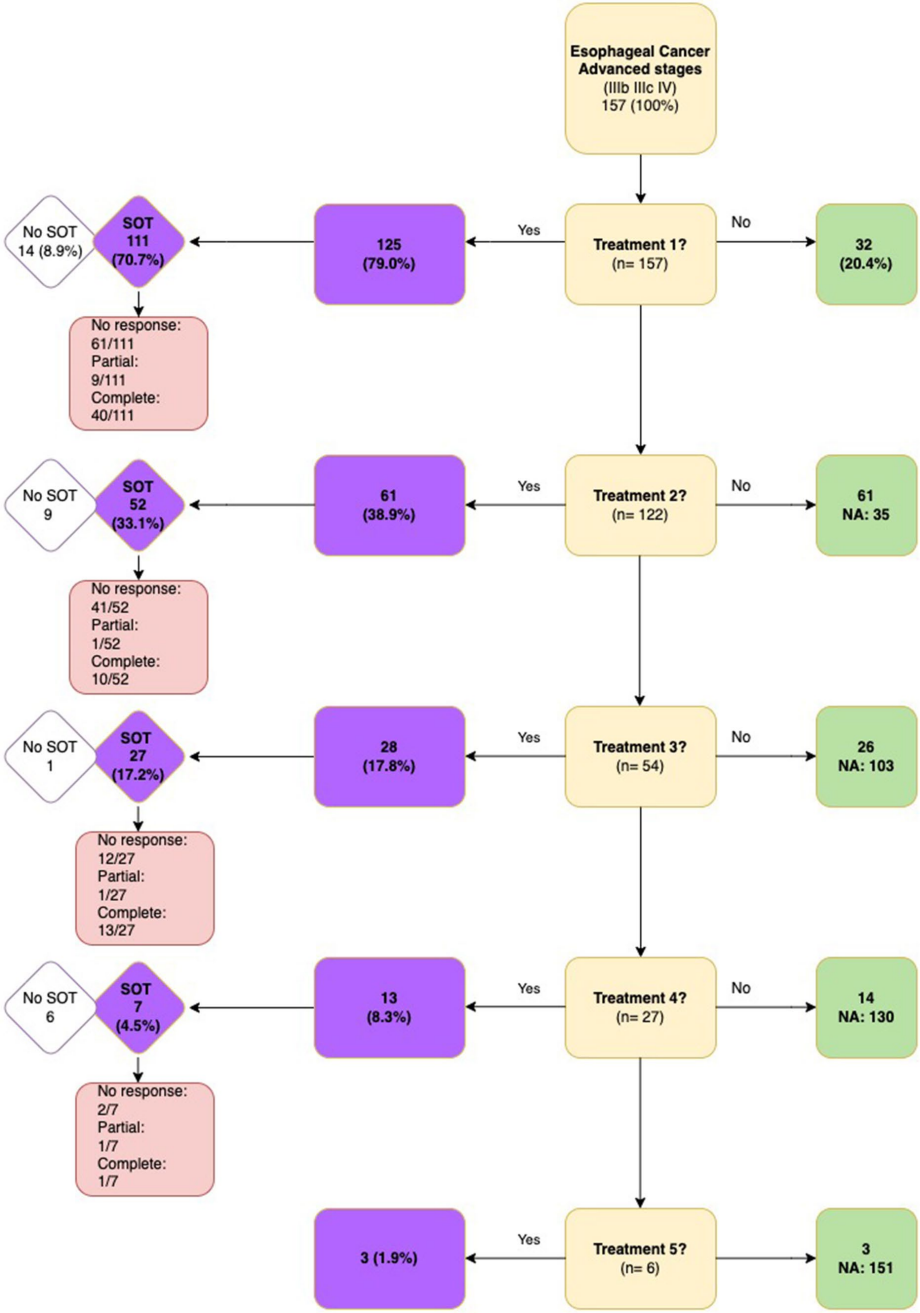
Treatment with SOT prescribed to advanced esophageal cancer patients at five hospitals, Spain, 2014–2016 (n = 157)

A brief outline of treatments with SOT administered to patients during the study period is presented in Fig. 1. Out of the 157 patients, 125 (79.6%) received at least one active treatment, whereas 32 (20.4%) patients did not receive any treatment; among them, 25 (15.8%) did not receive treatment due to their clinical condition, three patients refused treatment, and three died before any treatment decision. The median time between diagnosis and the initiation of the first treatment was 28 days. Patients who received the first treatment had a median age of 65 (IQR 57, 71) and an ECOG performance status of ≤ 2 in 88.5% of cases.

As the initial treatment, 10 out of 125 patients (8.0%) underwent surgery, with three undergoing trans-hiatal esophagectomy, two undergoing transthoracic esophagectomy, two undergoing laparoscopic esophagectomy, and three undergoing other procedures. Almost 90% of patients (n = 111, 88.8%) received SOT, while one-third (n = 43, 34.4%) were treated with radiotherapy. Only 40 patients (36.0%) showed a complete clinical response to SOT, while no response was observed in 61 (55.0%) cases and a partial response was seen in 9 cases. The treatment had to be interrupted in 69 patients (62.2%), either due to toxicity (n = 13), clinical deterioration (n = 17), or patient decision (n = 3). On average, patients received 4.4 cycles of SOT.

Almost 40% of patients (n = 61, 38.9%) received a second treatment, with 52 (85.2%) receiving SOT. Only ten patients of them had a complete response, while no response was observed in 41 patients, and one patient had a partial response. In 38 patients (62.3%), treatment had to be interrupted due to toxicity (n = 13), clinical status (n = 15), or patient decision (n = 2). Patients received an average of 3.8 cycles of second-line SOT.

As a third treatment, 28 (17.8%) patients received treatment, of which 27 (96.4%) received SOT. Only 12 cases reported a complete response, while 13 cases reported no response, and one case reported a partial response. In 20 cases (71.4%), the treatment had to be interrupted due to toxicity (n = 2), clinical status (n = 8), or patient decision (n = 1). Patients received an average of 3.9 cycles of SOT as their third treatment.

As the fourth treatment, 13 patients (8.3%) were treated, of whom 7 (53.8%) received SOT and six radiotherapy (46.2%) Only one case reported a complete response, while two cases reported no response, and one case reported a partial response. In two cases (15.4%), the treatment had to be interrupted due to toxicity (n = 1) or clinical status (n = 1). Patients received an average of 2.8 cycles of SOT. Lastly, regarding the fifth treatment line, only three patients (1.9%) received it.

In addition, eight patients (5.0%) participated in randomized clinical trials, including two in the BRIGHTER study (NCT01599650), one in the JAVELIN 300 study (NCT02625623), one in the TO-TAS-102-302 study (NCT02500043) as part of fourth-line therapy, and one in the WINTHER study (NCT01856296).

**Fig. 2:**
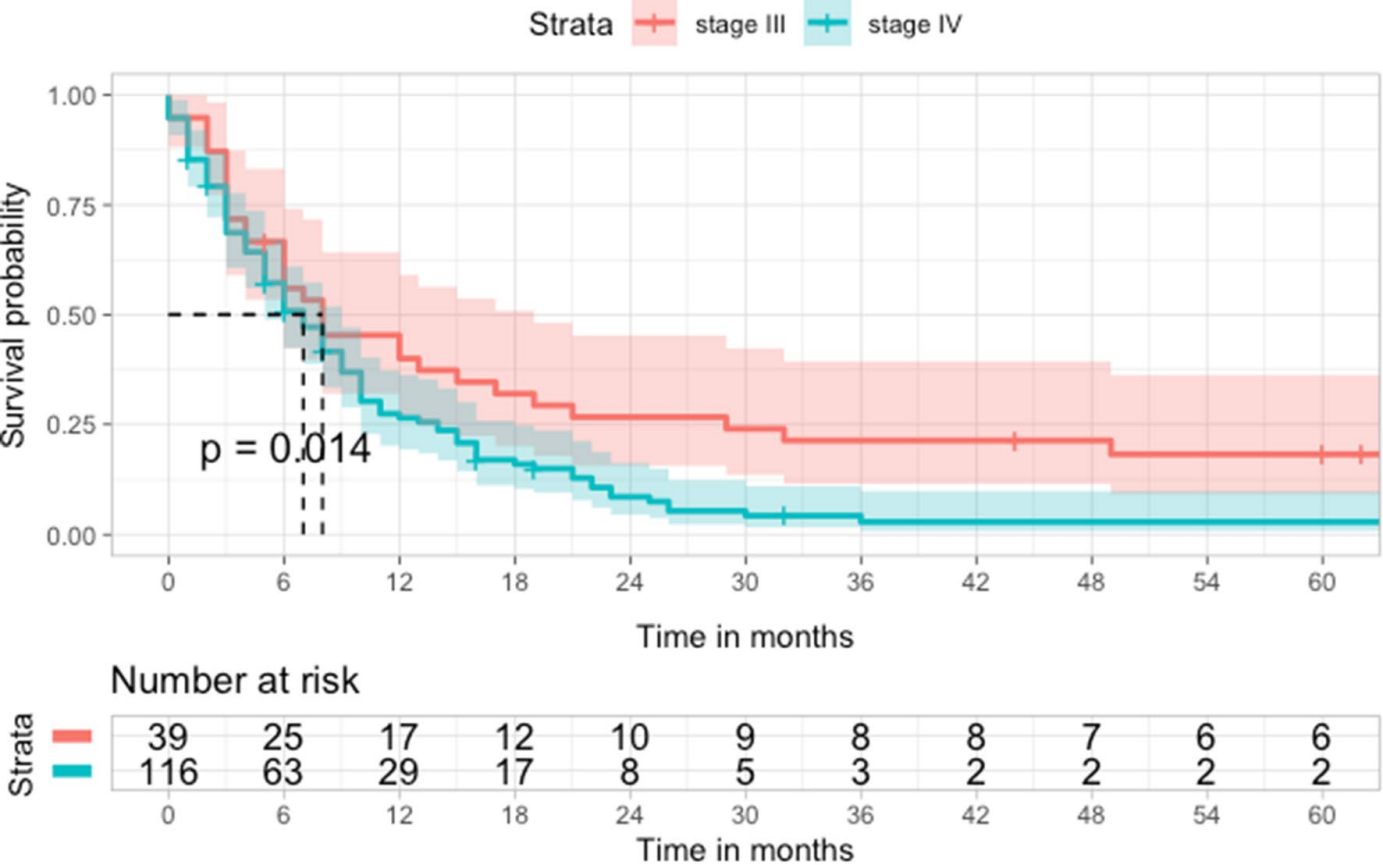
Kaplan–Meier survival curve of the overall survival pattern among advanced esophageal cancer patients (by stages) at five hospitals, Spain, 2014–2016 (n= 157)

During the study, the median follow-up was 6.0 months with an interquartile range (IQR) of 3.0 to 15.0 months. A total of 138 patients (87.9%) died, and the overall survival rate was very low, with 1-, 2-, and 5-year survival rates of 30.3% (95% CI: 23.7, 38.6), 13.3% (95% CI: 8.7, 20.2), and 7.2% (95% CI: 3.9, 13.2), respectively. When considering only stage IV cases, the survival rates were even lower. The 1-year, 2-year, and 5-year survival rates for stage IV patients were 27.5% (95% CI: 19.4, 36.2), 8.6% (95% CI: 2.8, 4.5), and 2.3% (95% CI: 1.0, 3.6), respectively. The median survival time was 7 months (95% CI: 6, 9), 8 months (95% CI: 6, 19) for stages IIIb IIIc and 7 months (95% CI: 5, 9) for stage IV (see Fig. 2).

The AFT model (Table 2) identified several factors that significantly affected survival time in advanced esophageal cancer patients. Clinical stage, ECOG performance status, receiving more than one line of SOT, and radiotherapy were found to accelerate the time to the event, with clinical-stage IV having the strongest effect (0.4 times shorter survival time compared to the baseline survival, p < 0.001). Conversely, an initial ECOG score of ≤ 2 was associated with an extended survival time by a factor of 2.18 (2.2 times longer survival compared to baseline, p = 0.04). Notably, age, sex, histology type, tumor location, or surgery were not statistically significant predictors of survival time.

**Table 2:** Determinants of survival time among advanced esophageal cancer patients at 5 hospitals, Spain, 2014–2016 (n = 157)

Determinants	TR	p-value
Age	1.0	0.2
Sex (male)	1.0	0.9
Hospital Sant Pau Taulí Donostia Reus Mataró	1 1.4 1.5 0.8 1.9	0.06
Histology adenocarcinoma squamous cell carcinoma other	1 0.6 0.5	0.12
Clinical stage (IV)	0.4	0.001***
Tumor location upper middle lower GEJ	1 1.6 1.2 1.1	0.6
ECOG PS (≤ 2)	2.2	0.04*
SOT more than 1 line (yes)	0.9	0.001***
Surgery first treatment(yes)	1.1	0.08
Radiotherapy first treatment (yes)	0.8	0.01*

^*^p < 0.05; ** p < 0.01, *** p < 0.001

*ECOG PS* *Eastern Cooperative Oncology Group Performance Status*, GEJ gastroesophageal junction, *PS* performance status, *SOT* systemic oncological treatment, *TR* time ratio

In the multivariate analysis (Table 3), only age < 65 years (OR 9.4, 95% CI 3.2, 31.4, p < 0.001) and hospital 3 (OR 0.2, 95% CI 0.0, 0.8, p < 0.01) remained significant predictors of receiving two or more lines of SOT.

**Table 3:** Predictors of more than 2 lines of SOT from logistic regression analysis among esophageal cancer patients registered at 5 hospitals, Spain, 2014–2016 (n = 157)

Predictors	AOR (95% CI)
Age (< 65 years)	9.4(3.2, 31.4)***
Sex (male)	1.6 (0.4, 6.7)
Hospital Sant Pau Donostia Mataró Reus Taulí	1 0.9 (0.2, 5.5) 0.2 (0.0, 0.8)** 0.0 (NA, 180.8) 1.6 (0.04, 6.1)
Histology adenocarcinoma squamous cell carcinoma other	1 1.3 (0.4, 3.9) 2.3 (0.3, 21.8)
Cancer stage (IV)	3.2 (0.9, 13.1)
ECOG PS ≤ 2	4.6 (0.7, 93.1)

*AOR* adjusted odds ratio, *CI* confidence interval, *ECOG PS* Eastern Cooperative Oncology Group Performance Status, *SOT* systemic oncological treatment

*P < 0.05; **P < 0.01, ***P < 0.001

Table 4 displays the appropriateness indicators of treatment in advanced esophageal cancer patients at five hospitals in Spain from 2014 to 2016. Approximately 18.0% (20/111) of patients received their last treatment cycle during the last month of their life, and 9.0% (10/111) it in the final two weeks before death. Additionally, 3.6% (4/111) of patients were prescribed a new line of chemotherapy during the last month of life. Moreover, 13.0% of patients with a registered ECOG performance status of ≥ 3 were still treated with anticancer drugs. We observed that 29.3% of patients (46/157) were never referred to palliative care services. Among those who accessed it, 34.2% (38/111) were referred during the last month of life, and 6.3% (7/111) in the final three days before death. Additionally, more than two-thirds of patients (97/139, 69.8%) died in the hospital.

**Table 4:** Appropriateness indicators of advanced esophageal cancer patients at five hospitals, Spain, 2014–2016 (n = 157)

Area	Indicators	Study results
Overuse of chemotherapy very near death	(1) Chemotherapy in the last 14 days of life (2) Chemotherapy in the last 30 days of life	9.0% (10/111) 18.0% (20/111)
	(3) Starting a new line of chemotherapy in the last 30 days of life	2.5% (4/157)
Underuse or late use of palliative care services, or death in an acute-care setting	(4) The proportion of patients with late-stage disease who did not access to palliative care	29.3% (46/157)
	(5) First access to palliative care < 30 days before death (6) First access to palliative care < 3 days before death	34.2% (38/111) 6.3% (7/111)

For all indicators, we present all time periods validated by expert panels (see reference in the text) and commonly used in the literature

## Discussion

In this study we assessed the appropriateness of SOT provided to patients diagnosed with advanced EC across a group of participating hospitals in Spain. In doing this, we analyzed the clinical characteristics and treatments administered to these patients followed during a period of 5 years.

Our results confirm that advanced EC patients have a very poor prognosis because the overall 1-, 2- and 5-year overall survival rates were 30.3%, 14.0%, and 7.1%, and the median survival was 7 months (stage IV 5-year overall survival 2.3%). These findings are very concordant with those reported elsewhere, i.e. by the US Surveillance, Epidemiology, and End Results (SEER) Program (5-year relative survival rate for distant stage = 5.0%) [39] and also consistent with prior international studies that have reported a 3-year survival rate less than 10% in advanced stages (stage IV: Canada 3.82 0.39–7.26, Denmark 3.28 1.24–5.32, Ireland 5.12 1.06–9.17, UK 3.69 2.71–4.67) [6]. Nevertheless, it is important to highlight that to date there are few studies that publish survival data by clinical stage, and those that do so are from high-income countries (i.e., ICBP SURVMARK-2 project [6], Cancer Research UK).

We found that almost 80% of patients received SOT, despite limited evidence on their effectiveness in these cases [27]. For example, European guidelines recommend first or second line treatments only for selected cases [37, 40], yet our study found that almost 18% of patients received a third line, 8.3% a fourth, and 2% a fifth treatment, without participation in clinical trials (Fig. 2). Furthermore, almost 20% of patients received chemotherapy in the last month of life, and some even started a new line of chemotherapy in this period (Table 4). These findings suggest a potential overuse of SOT, which could harm patients more than benefit them [41].

Almost 30% of patients did not have access to palliative care, and 34.2% and 6.7% were referred only during the last month of life or 3 days before death, respectively. This underuse of palliative care contradicts authorized claims for the early provision of palliative services for advanced cancer patients [42–45].

Finally, our results detected a high variability in treatments administered across hospitals, once adjusted by the other factors. Clearly, some hospitals greatly differ in the number of SOT lines being administered. Additionally, receiving more than one line of SOT was statistically associated with a worse prognosis and accelerated time to death. While we did not find comparable data from previous studies on esophageal cancer, a similar pattern was observed in advanced non-small cell lung cancer [46]. These findings suggest that receiving multiple lines of SOT may not always benefit patients and could potentially harm them. However, the small numbers in these analyses advise having caution about this interpretation.

Overall, our study highlights the need for a more judicious use of SOT and earlier access to palliative care for advanced EC patients. Further research is needed to better understand the factors contributing to the administration of SOT to patients with such poor prognosis on a short-term basis and therefore to identify strategies to improve the quality of provided care.

This study has some limitations that need to be acknowledged. The main limitation is their retrospective design, in which missing and heterogeneous or inconsistent data in the medical record can be common. We were able to minimize some of these issues due to the experience and specialized training of data collectors. However, we observed that almost 90% of patients had registered a good performance status (ECOG 0, 1 or 2) which probably does not reflect the true functional capacity of many of these advanced patients even though they were treated. As it has been repeatedly reported, measuring the PS by treating doctors is open to bias because they are prone to overestimate it [44]. A second limitation is that the five included hospitals may have different services, including radiotherapy or palliative care, which could influence the therapeutic decisions that have been taken. Another important aspect to remark is that although we used time-dependent analyses to minimize immortal time bias [47], it is essential to recognize that no study design can completely eliminate it. Therefore, caution should be reminded when interpreting these results. Finally, we must remark that the development of effective quality indicators for assessing the appropriateness of EOL care presents inherent challenges [48]. The available evidence base is limited, and there exists a lack of consensus among both experts and patients regarding the definition of optimal care during this crucial period [49]. Moreover, the proportion of patients undergoing cancer-directed treatment at the end of life shows significant variation and has been widely reported in numerous publications [50]. Unfortunately, we were not able to identify in the medical records patients’ preferences nor physician opinions to justify the decisions taken.

To our knowledge, this study is the first reported multicenter, comprehensive, and validated data collection with 5 years of follow-up on advanced esophageal cancer aimed to assess the appropriateness of provided care. Based on our results, we conclude that a high proportion of advanced EC patients are overtreated with SOT and do not benefit enough from palliative care services. This study provides important insights into the appropriateness of treatment in advanced EC, and furthermore, has many implications for understanding the variability of local practices and giving valuable opportunities for improvement.

## Data Availability

The data supporting the findings of this study are available within the article and its supplementary information files. Additionally, raw data and materials are available upon request from the corresponding author, Marilina Santero, upon reasonable request and with permission from the relevant data owners, ensuring compliance with confidentiality and ethical considerations.

## Appendix 1

### General characteristics of selected hospitals

**Table:**

Name of the centre	City	Beds	Centre class	Functional dependency	Teaching accreditation	SNS agreement
Hospital de la Santa Creu i Sant Pau	Barcelona	644	General hospitals	Privates	Yes	Public use network (RUP)
Corporoació Sanitària Parc Taulí	Sabadell	861	General hospitals	Other public entities or organisms	Yes	Public use network (RUP)
Hospital Universitario Donostia	Donostia/San Sebastián	1034	General hospitals	Health services and institutes from autonomous communities	Yes	No agreement
Hospital Universitari de Sant Joan de Reus	Reus	313	General hospitals	Municipality	Yes	Public use network (RUP)
Hospital de Mataró	Mataró	402	General hospitals	Other public entities or organisms	Yes	Public use network (RUP)

Source: Gobierno de España, Ministerio de Sanidad (2020). “Catálogo Nacional de Hospitales 2021” https://www.sanidad.gob.es/ciudadanos/prestaciones/centrosServiciosSNS/hospitales/docs/CNH_2021.pdf

Name of the centre: name of the centre that appears in the register of the Autonomous Community, Cities with Statute of Autonomy or Ministry of Defence, which appears in its registration and authorization and which has been transferred to REGCESS

Beds: installed beds are considered, at the date of data collection, those that constitute the fixed staff of the hospital and that are ready to be used, although some of them may, for various reasons, not be in service on that date

Centre class: refers to any of the classes defined in the Classification of health centres, services, and establishments to which it belongs, according to Annex I of Royal Decree 1277/2003, which establishes the general bases for the authorization of centres, services, and health establishments. It defines Hospitals (internment centers) as health centers intended for the specialized and continuous care of patients in an internment regime (at least one night), whose main purpose is the diagnosis or treatment of patients admitted to them, without prejudice that they also provide care on an outpatient basis

Functional dependency: the functional dependency of a center is understood to be the body or legal entity on which it depends, that is, the natural or legal person who exercises domain or jurisdiction, hierarchical or functional, more immediate over the health establishment, regardless of its form of management. Both the definition and the current values correspond to those defined in Order SCO/3866/2007, of December 18, which establishes the content and structure of the General Registry of health centres, services, and establishments of the Ministry of Health and Consumption

Teaching accreditation: reports that the center has the teaching accreditation that enables it to provide specialized postgraduate health training and that at the time of data collection had professionals in training

SNS agreement: reports whether a private dependency center provides services to the National Health Service (SNS), regardless of whether the agreement is partial or substitute

## Declarations

### Patient and public involvement

Neither the patients nor the public were not involved in the design, conduct, reporting or dissemination plans of this research.
